# School lives of adolescent school students living with chronic physical health conditions: a qualitative evidence synthesis

**DOI:** 10.1136/archdischild-2022-324874

**Published:** 2022-12-02

**Authors:** Bethan K C Spencer, Judy Wright, Kate Flemming, David Cottrell, Simon Pini

**Affiliations:** 1 Leeds Institute of Health Sciences, University of Leeds, Leeds, West Yorkshire, UK; 2 Department of Health Sciences, University of York, York, North Yorkshire, UK

**Keywords:** qualitative research, adolescent health

## Abstract

**Objective:**

Assess the existing evidence base in order to synthesise the current qualitative findings for the impact of chronic health conditions on the school lives of young people.

**Design:**

Qualitative evidence synthesis using thematic synthesis.

**Patients:**

Young people aged 11–18 years with a chronic health condition from one of the following groups: oncology, cystic fibrosis, diabetes, asthma, rheumatology, neuromuscular, colorectal, chronic pain, allergies and dermatology.

**Outcome measure:**

Qualitative findings and discussions present in included studies formed the data for the thematic synthesis.

**Results:**

From a search identifying 19 311 records, a sample of 35 papers were included. The included papers represented 15 countries and primarily employed interviews as part of data collection. Thematic synthesis resulted in six themes: ‘keeping up/catching up/missing out/looking forward’; ‘identity’; ‘relationship with peers’; ‘normality and difference’; ‘autonomy’; ‘relationships with staff’.

**Conclusions:**

Thematic synthesis highlighted the commonalities, rather than divergence, of issues for young people across different chronic conditions. Policies need to be based on the experiences of the people they aim to provide for, and while attendance and attainment remain important, there is clearly more that needs to be considered when gathering data, designing interventions and developing policies to support this population. It may also be advisable for clinical professionals to include the broader psychosocial aspects of school life in discussions and plans to support young people with long-term conditions.

**PROSPERO registration number:**

CRD42021278153.

**WHAT IS ALREADY KNOWN ON THIS TOPIC**

- The majority of research into education outcomes for young people with long-term conditions is disease specific and focuses on attendance and attainment. Qualitative research in this topic is disparate and requires synthesis to establish commonalities.

**WHAT THIS STUDY ADDS**

- Thematic synthesis resulted in six themes reflecting commonalities in the findings of studies from diverse health conditions: keeping up/catching up/missing out/looking forward, identity, relationship with peers, normality and difference; autonomy and relationships with staff.

**HOW THIS STUDY MIGHT AFFECT RESEARCH, PRACTICE OR POLICY**

- Policy and practice need to focus on diverse aspects of school life beyond attendance and attainment. The commonalities in the findings also suggest that cross-condition working is the desirable direction for future research and policy development.

## Introduction

Chronic physical health conditions are defined as those requiring ongoing management over a period of years that can be controlled, but not cured, with the use of medication and other therapies.^1 2^ However, there is inconsistent use of terminology across the field when definitions are created through the lens of different specialisms.^3^ Taking into account the variety of definitions, it is estimated that 13%–27% of children are affected by chronic conditions.^4^ In the UK, this figure is approximately 1–1.7 million.^5–8^

Most research into the impact of chronic health conditions on education focuses on quantitative outcomes of school attendance and attainment and concludes that outcomes are negatively impacted by a chronic health condition to varying degrees.^9–17^ For example, studies conducted in childhood epilepsy, diabetes and asthma all showed relative deficits in educational attendance or attainment.^10–12^

However, attainment and attendance do not tell us how school life is experienced by young people with chronic conditions. The aim of this review of qualitative research was to establish what current research tells us about the experience of school life for young people with a chronic condition in 1 of 10 clinical groups. The review focuses on the UK age range for high school, which is 11–18 years. The results inform the initial stage of a wider project (the INSCHOOL project) to develop impactful policies, support and screening assessments that are grounded in the experiences of young people.

## Method

### Research question

What do high school pupils say about the impact their health condition has on their school lives?

### Design

A qualitative evidence synthesis using thematic synthesis.^18^

### Search strategy

In September 2021, comprehensive searches of 13 databases and a search engine were conducted for qualitative studies on the impact of chronic or life limiting health conditions on high school lives (see online supplemental file 1).

10.1136/archdischild-2022-324874.supp1 Supplementary data

Searches were developed for: adolescents, school settings, 10 chronic conditions, outcomes covering quality of life, education and psychological, and qualitative methods. Subject headings and free text words were identified from relevant papers by JW and SP. The filters described by DeJean *et al* 19 were applied with minor adaptations for qualitative papers (online supplemental file 1 reports full search strategy). Bibliographies were scrutinised for further studies. Results were deduplicated in EndNote (X9) before being transferred to Covidence for screening and data extraction.

### Inclusion criteria

- Qualitative methods.
- First-hand accounts from young people aged 11–18 years.
- Data related to school life.
- The INSCHOOL project is working directly with young people and clinicians from children’s services in a parallel qualitative project in a large urban setting in the north of England. To align with this project, and to represent diversity of visibility, severity and predictability, the following health conditions have been included in the current review:
  - Oncology.
  - Cystic fibrosis.
  - Diabetes.
  - Asthma.
  - Rheumatology.
  - Neuromuscular.
  - Colorectal.
  - Chronic pain.
  - Allergies.
  - Dermatology.

### Study screening methods

Title screening was conducted by BKCS, with marginal cases discussed with SP, followed by abstract screening by both. Full texts were then reviewed by BKCS and SP who judged inclusion independently before resolving discrepancies. Appraisal was guided by pragmatic assessment of content and utility of findings related to the research question. The school lives of young people with chronic health conditions did not have to be the primary focus of the research, provided that the findings were relevant to the review.

### Appraisal items

CASP Qualitative Studies Checklist^20^ was used to assess methodological quality. BKCS and SP applied this checklist alongside data extraction and resolved uncertainties. A-priori cut-off criteria were not set, as the review aimed for inclusivity of relevant findings. Papers with multiple answers of ‘no’ or ‘unsure’ were reviewed with for exclusion.

### Data extraction

BKCS and SP conducted data extraction independently and resolved discrepancies. Essential contextual information and methodological detail were extracted using a study-specific data extraction form. In preparation for thematic synthesis, results and discussion sections were the primary source of findings, alongside quotes from participants and authors. These sections were approached line by line and scrutinised for relevant content.

### Synthesis methodology

Thematic synthesis drew together findings across studies into descriptive and analytic themes. Analysis was inductive and sought to critically approach extracted data independent of original author interpretation. This allowed novel concepts and interpretations to arise. Synthesis began using a group of four papers to establish a first draft of the thematic framework. These papers were identified during full-text review as most relevant to the research question. The initial framework was then applied to remaining papers in an iterative process of refinement through discussion of negative cases and emerging concepts.

## Results

### Study selection results

**Figure 1:**
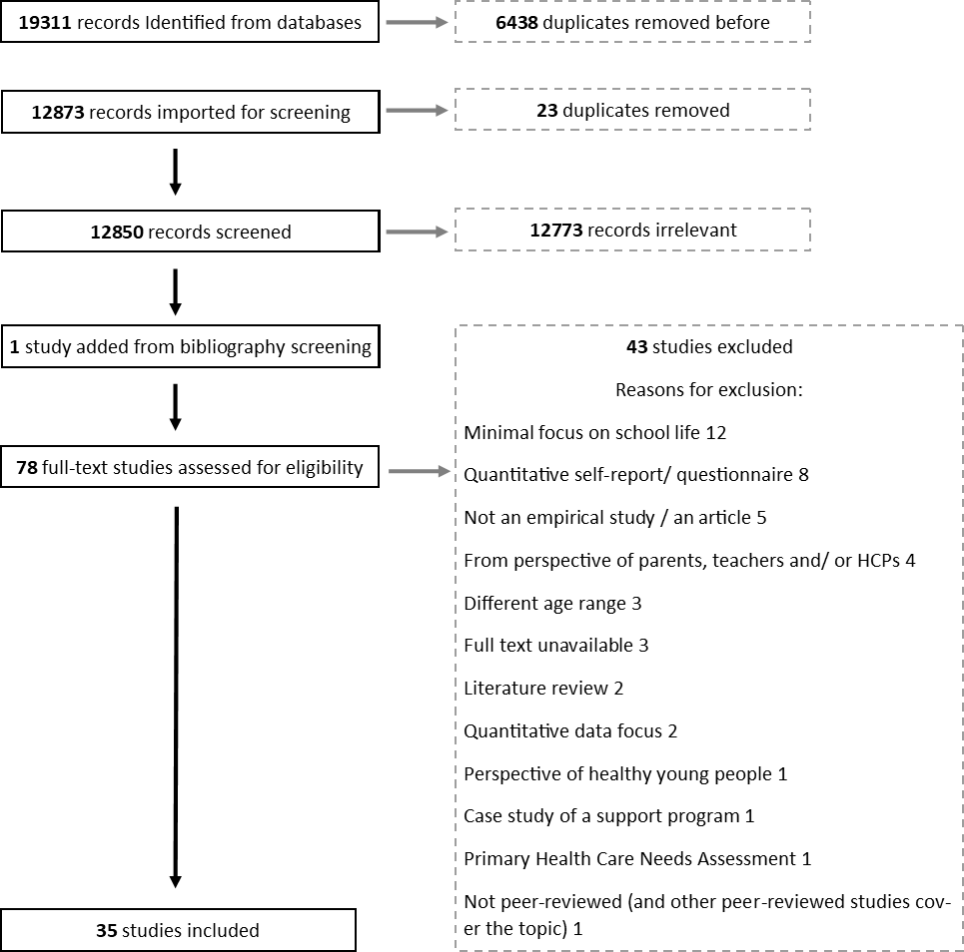
PRISMA diagram. PRISMA, Preferred Reporting Items for Systematic Reviews and Meta-Analyses.

From a search identifying 19 311 records, 35 papers were included. The Preferred Reporting Items for Systematic Reviews and Meta-Analyses diagram details how studies were excluded (figure 1).

### Study characteristics

Included papers represented 15 countries spanning 1995–2021. The majority (32/35 studies) employed interviews. Details are presented in online supplemental file 2.

10.1136/archdischild-2022-324874.supp2 Supplementary data

### Appraisal results

No a-priori cut-offs were used. Following review of the CASP checklist with the research team, it was decided no studies needed to be excluded based on methodology. Full study appraisal can be found in online supplemental file 3.

10.1136/archdischild-2022-324874.supp3 Supplementary data

### Synthesis output

Assessment of the four most relevant papers produced 16 descriptive themes, which were developed into an initial framework of six overarching analytical themes. These were further refined through iterative application to the remaining papers. The six analytical themes remained stable, with each paper adding richness to the themes rather than producing new thematic directions (thematic contribution displayed in online supplemental file 4).

10.1136/archdischild-2022-324874.supp4 Supplementary data

Thematic synthesis resulted in six themes:

- Keeping up/catching up/missing out/looking forward.
- Identity.
- Relationship with peers.
- Normality and difference.
- Autonomy.
- Relationships with staff.

Each theme is detailed with reference to relevant participant/author extracts (further extracts can be found in online supplemental file 5).

10.1136/archdischild-2022-324874.supp5 Supplementary data

#### Keeping up/catching up/missing out/looking forward

Absence was a key aspect of managing education.^21–30^ Maintaining education in spite of absences was important to young people and staying connected with school benefitted students.^26 31–35^ Lakeman highlighted ‘*keeping up*’, ‘*catching up*’ and ‘*missing out’*, which are core concepts reflected in 31 of the papers and highlighted by shortened lessons, leaving early or missing school for treatment.^24 27 28 36^ However, even when able to attend school, difficulty concentrating, fatigue and pain made remaining engaged in lessons a struggle.^23 26 36–39^ ‘*Missing out*’ was not only related to school work, but also school trips, social and physical activities^27 33 38 40^ and could affect social involvement, leaving young people ‘*out of the loop*’.^21 22 26 27 29 36–38 41–43^

Underlying the ‘*burden*’^36^ of catching up was concern about falling behind in school and the potential impact on attainment.^21 23–25 33 36 38 39 44 45^ Studies reported concerns related to pressure from themselves and others,^23 39 46^ a lack of confidence,^23^ a lack of provisions^31^ or feeling excluded from the curriculum.^27^ Exams and grading were often benchmarks of ‘normal’ progress or keeping up with peers, as well as a means of shaping the future.^46^ Young people expressed concerns they would be lacking certain skills as a result of missed school.^33 44 47^ However, many had focused long-term goals for their education and career, often influenced by their experience of living with a health condition.^32 38 47^ There were examples where health experiences were constructively incorporated into future plans and others where these plans were in defiance of the limitations they had faced.^32^

#### Identity

Studies suggested the importance of acknowledging that secondary school years are a time when self-identity develops, particularly in context of the school environment.^32 48^ The emergence of various identities as an adolescent and student can remain separate from, or intertwined with a young person’s chronic health condition.^32 47–49^ While Cotter (2016) found a young person’s identity as a student develops separately from their chronic condition identity (cystic fibrosis), other studies found young people’s identities merge with their condition, in more profound cases leading to a sense of losing a former self, or life being ‘*ruled by illness*’.^50^ ^22 26 49 50^ For some, this meant distancing themselves from condition labels, the ‘*sick-patient*’ role and ‘*personal tragedy*’^47 49^ and wanting to ‘*remain the same but changed*’.^46^

Some young people referred to ‘*visible differences*’ such as hair loss,^25 33 36^ weight loss,^32^ mobility difficulties^38^ and scars^32^ as a result of their condition.^32 38 43^ Such changes in appearance could shape school experiences by providing a ‘*before and after*’ during reintegration.^46^ Visible changes could result in unwanted attention and altered peer dynamics, and young people reported hiding visible differences.^24 36 41 46^ To compound this issue, several studies reported the negative impact of school staff projecting a ‘*disabled identity*’^38^ or ableist attitudes onto the young people.^38 47 50^

#### Relationships with peers

Peer groups are a central aspect of school life and an important source of practical and emotional support.^21 22 25 26 28 29 31 32 35 37 38 40 41 43–46 48 51 52^ Young people express a desire for ongoing contact and social acceptance within school.^24 25 35 46 47^ Exclusion from social groups could result in feelings of isolation, alienation and invisibility.^22 26 31 36 38 47^ Young people highlighted the distressing effect of being ignored and a lack of understanding and empathy from peers^21 27 31 53^ who could see conditions as ‘*disgusting’* or ‘*strange’* 50 and, in some cases, result in physical and verbal bullying.^25 27 32 40 41 43 50^ Young people with health conditions were often the focus of increased attention from peers and found themselves explaining their condition, sometimes repeatedly, and subject to curiosity and questioning.^27 36 47 48 54^ Some saw this as a chance to help others understand and to ensure correct information was shared, but many found it frustrating, exhausting and ineffective,^21 42 48 54^ especially coping with disproportionately emotional or negative responses.^23 25 38 41 54^

#### Normality and difference

Overall, young people expressed a wish to be treated the same as healthy peers and to be perceived as normal,^24 32 37 41 49 53^ although this was often not the case.^23 25 31 38 54^ There was also tension between a desire for normality and a need for some special consideration.^22 38 46 49^

For many, school represented a stabilising environment with the potential for them to cultivate a return to normality after diagnosis^25 32 33 43^ or redefine normal in their own way.^33 45^ However, there are many challenges to the desire for normality in school and multiple studies referred to young people’s sense of difference or ‘abnormality’.^22 24 38 40 43 45 47 51 54^ Some approached this by concealing their condition in order to ‘*act normally*’ or ‘*pass as normal*’.^37 47 53^ Ferguson and Walker^32^ highlighted this approach to normality meant some young people ‘*slipped under the radar*’.

#### Autonomy

Studies reported feelings of reduced independence at a time when it was an important developmental milestone^33 38 45 49^ and school was identified as providing an opportunity to regain independence and a sense of freedom.^45^ Young people reported finding self-management, such as carrying and taking medication in school, a means of gaining independence, but this has been described as a challenging responsibility.^37 38 41 42 47 50 51^ Young people wished to have easy access to medication, and the option of private safe spaces.^37 40 47 55^

Disclosure and controlling the narrative of their health condition was a significant factor in autonomy within school.^33 38 41 42 48 54^ Some young people chose not to disclose their health condition, or only disclosed when necessary,^24 43 48^ whereas others were comfortable with disclosure.^29 43 54^ Being involved in the process of sharing their ‘story’ was an important aspect of managing chronic health within school,^26 27 33 54^ as young people could have some control of their own information, be able to shape a positive outlook and minimise rumours.^33 54^

In some studies, young people wanted to be heard in decision making and expressed a preference for person-centred practice, which was often absent.^26 27 38^ Studies revealed that young people valued having knowledge and understanding of their condition, with some seeing educating others as empowering in the school environment.^24 41 55^

#### Relationships with staff

Teachers having awareness and, in some cases, experience of their condition was an important factor in feeling supported at school.^27 28 31 37 38 50 55^ Two studies suggested that more education for school staff on how to manage the health needs of young people is needed, as many staff do not know how to ‘deal with them’.^29 55^ Young people valued empathetic teachers, those who reacted supportively to disclosure and were willing to make adjustments.^24 27 31 32 38 50 51 53^ This could be enhanced by having one key member of staff offering support.^24 25 27^

Conversely, teachers lacking awareness and empathy had a negative impact on students’ school lives.^27 38 50^ In some cases, teachers considered provisions to be ‘*so much fuss*’^50^ and young people faced disbelief for the impact of their conditions, especially when there were few visible aspects.^30 40 50^ This could lead to avoiding asking for help, feelings of isolation and concealment of symptoms in order to avoid stigma and judgement from teachers.^22 40 53^

The quality of communication and maintaining contact was important to students and affected the extent to which they felt cared about.^34 35 46^ In some studies, the communication between healthcare professionals and teachers was inadequate or absent, meaning parents or motivated individual teachers assumed responsibility for this role.^27 37 55^

## Discussion

This review has highlighted important themes present in the school experiences of young people across diverse chronic conditions. Themes were developed from papers focusing on different chronic conditions and highlight the commonalities, rather than divergence, of issues for young people regardless of condition. However, more focused work needs to be conducted in this area to highlight where specific conditions may result in specific challenges. It may be advisable for professionals and services, whether clinical or educational, to include the broader psychosocial aspects of school life in discussions and plans to support young people with long-term conditions. It is feasible that attending to the breadth of these issues has potential to improve the long-term educational, psychosocial and physical outcomes for this population, but more research will be needed in this area.

Policies need to be based on the experiences of the people they aim to provide for,^56^ and while attendance and attainment remain important, there is clearly more that needs to be considered when gathering data, designing interventions and developing policies to support this population. Data related to important holistic aspects of the school lives of young people, such as peer relationships, autonomy and normality, are not gathered and integrated into education datasets. This is further complicated for young people with chronic health conditions, as there is also a lack of integration of health and education data. Therefore, if policies and markers of progress are driven and evaluated by data, but the datasets are missing holistic psychosocial and health information, there is an increased chance of marginalising young people with chronic conditions.

More research is needed to further investigate the holistic and psychosocial needs of young people with chronic health conditions at school. Holistic data need to be gathered and integrated into education datasets to enable more informed policy making, more accurately targeted interventions and more nuanced evaluation of outcomes.

### Strengths and limitations

This review uniquely synthesised qualitative evidence across chronic conditions and presented important insights into the school experiences of this population. However, the intentional breadth of this review means there are several areas requiring more focused future research and elaboration.

## Data Availability

All data relevant to the study are included in the article or uploaded as supplementary information.
