# Supplementary material for: School lives of adolescent school students living with chronic physical health conditions: a qualitative evidence synthesis
Source: Arch Dis Child. 2022 Dec 2;108(3):225–9. doi: 10.1136/archdischild-2022-324874 (PMC9985755; doi:10.1136/archdischild-2022-324874)
Supplement: Supplementary data [file archdischild-2022-324874supp001.pdf]

INSCHOOL Search Strategy

12/09/2021

Sources of published and grey literature

Content Type	Source Name and platform (or URL)
Published literature only	CINAHL (EBSCOhost)
	Cochrane Database of Systematic Reviews (Wiley) Issue 9 of 12, September 2021
	Core Collection (Web of Science) SCI-EXPANDED 1900+, SSCI 1900+, and ESCI 2015+ searched simultaneously.
	Education Abstracts (H.W. Wilson) (EBSCOhost)
	Ovid MEDLINE(R) ALL 1946 to September 16, 2021
Published and grey literature	Embase Classic+Embase (Ovid) 1947 - 2021 September 16
	ERIC (EBSCOhost)
	Google Scholar https://scholar.google.com/
	Social Care Online https://www.scie-socialcareonline.org.uk/
Grey literature only	Core Collection (Web of Science) CPCI-S 1900+ and CPCI-SSH 1900+ searched simultaneously.
	EThOS E-Theses Online Service https://ethos.bl.uk/Home.do

Ovid MEDLINE(R) ALL <1946 to September 10, 2021>

1 exp qualitative research/ 67321

2 "Surveys and Questionnaires"/ 508848

3 exp interview/ 29874

4 interview*.tw,kf. 393491

5 qualitative.tw,kf. 265881

6 questionnaire*.tw,kf. 571774

7 survey*.tw,kf. 721796

8 ethnolog*.mp. 171458

9 ethnograph*.mp. 12147

10 ethnonursing.af. 125

11 phenomenol*.af. 29629

12 (grounded adj (theor$ or study or studies or research or analys?s)).af. 13324

13 (emic or etic or hermeneutic* or heuristic* or semiotic*).af. 19853

14 (data adj1 saturat*).tw,kf. 1545

15 participant observ*.tw,kf. 4950

16 action research.mp. 4761

17 (cooperative inquir* or co-operative inquir*).mp. 78

18 ((purpos* adj4 sampl*) or (focus adj group*)).af. 75046

19 (life world or life-world).mp. 366

20 conversation analys?s.mp. 780

21 personal experience*.mp. 14906

22 theoretical saturation.mp. 247

23 cluster sampl*.mp. 8253

24 observational method*.af. 855

25 content analysis.af. 33730

26 ((discourse* or discurs*) adj3 analys?s).tw,kf. 2707

27 narrative analys?s.af. 1527

28 or/1-27 [Adapted De Jean Qualitative studies filter] 1949143

29 (Chronic* adj2 (disease* or health or illhealth or condition or illness*)).tw,kf. 261058

30 (chronic* adj2 pain*).tw,kf. 63122

31 (long-term adj2 (disease* or health or illhealth or condition? or illness* or pain*)).tw,kf. 26499

32 asthma?.tw,kf. 157841

33 (broncho* or bronchial*).tw,kf. 197052

34 (cancer* or carcinoma* or neoplas* or adenocarcinoma* or malignan* or tumo?r* or sarcoma*).tw,kf. 3654018

35 (cystic* adj3 fibros*).tw,kf. 47929

36 (fibrocystic adj8 disease adj8 pancreas).tw,kf. 214

37 diabet*.tw,kf. 690969

38 eczema*.tw,kf. 20977

39 (rheumatoid* adj2 arthriti*).tw,kf. 117161

40 rheumati*.tw,kf. 61294

41 (muscular* adj2 (syndrome* or condition? or disease*)).tw,kf. 3075

42 (neuromusc* adj2 (syndrome* or condition? or disease*)).tw,kf. 7908

43 Motor neuron disease.tw,kf. 5235

44 ("peripheral neuropathy" or "muscular dystrophy" or fibromyalgia).tw,kf. 55253

45 ((bowel* or colon* or intestinal or rectal* or anal) adj2 (disease? or syndrome? or condition?)).tw,kf. 90759

46 ((skin or dermat*) adj2 (disease? or condition? of syndrome?)).tw,kf. 37404

47 (allergy or allergies or allergic reaction? or hypersensitiv*).tw,kf. 175885

48 exp Asthma/ or Bronchial Spasm/ or exp Bronchoconstriction/ or Bronchial Hyperreactivity/ or Respiratory Hypersensitivity/ 147212

49 exp Neoplasms/ 3532612

50 exp Diabetes Mellitus/ 454034

51 exp Cystic Fibrosis/ 36935

52 exp Arthritis, Rheumatoid/ or exp Rheumatology/ 123976

53 exp Neuromuscular Diseases/ 321561

54 exp Intestinal Diseases/ 659941

55 exp Hypersensitivity/ 358007

56 Chronic disease/ 270493

57 exp Skin Diseases/ 1069704

58 exp Chronic Pain/ 17627

59 or/29-58 [10 chronic diseases] 7488197

60 (classroom? or class-room?).tw,kf. 19567

61 (school? not (medical school? or "school of medicine" or "nursing school? of school of nursing")).tw,kf. 246265

62 "School-based".tw,kf. 15176

63 Schools/ 43312

64 School Health Services/ 17823

65 (secondary adj2 education).tw,kf. 4057

66 or/60-65 [Schools] 269837

67 Adolescent/ 2120701

68 Child/ 1774320

69 (teen* or adolesce* or youth).tw,kf. 389409

70 (school* adj2 (student? or pupil?)).tw,kf. 25176

71 (("11" or "12" or "13" or "14" or "15" or "16" or "17" or "18") adj3 (year? or yr?) adj3 (old or age?)).tw. 438839

72 students/ or student dropouts/ 68102

73 or/67-72 [Adolescents] 3203822

74 Educational Status/ or Academic Success/ 55523

75 Social Participation/ 2880

76 *"Quality of Life"/ 99353

77 (Cogniti* or Social* or Attendance or Academic* or Psychosocial or psycho-social).ti. 331725

78 "Quality of life".ti. 76626

79 (view* or attitude* or perspectiv* or presentee*).ti. 273586

80 (attain* or adaptation or absen* or potential or achieve* or relationship?).ti. 619940

81 exp Emotions/ 267530

82 (emotion* or contentment or enjoy* or impact* or feel* or normal* or safe* or behavio* or functioning).ti. 1063789

83 (experience* or outcome?).tw,kf. 2930401

84 or/74-83 [outcomes] 4989224

85 84 and 28 and 59 and 66 and 73 [Adolescents with 10 conditions AND outcomes AND Qualitative studies] 4017
