# Supplementary material for: School lives of adolescent school students living with chronic physical health conditions: a qualitative evidence synthesis
Source: Arch Dis Child. 2022 Dec 2;108(3):225–9. doi: 10.1136/archdischild-2022-324874 (PMC9985755; doi:10.1136/archdischild-2022-324874)
Supplement: Supplementary data [file archdischild-2022-324874supp002.pdf]

Author (date)	Title	Country the study is based in	Relevant participant details	Data collection method	Key findings
An and Lee (2019)	Difficulty in returning to school among adolescent leukaemia survivors: A qualitative descriptive study	South Korea	14 participants<br>14-22 y.o.<br>Leukaemia	Semi-structured interviews<br>Thematic analysis	Feelings of alienation from friends<br>Difficulty in studying<br>Stuck being different from others<br>Apologetic feelings for family<br>Feelings of having an uncertain future
Bessel (2001)	Children surviving cancer: Psychosocial adjustment, quality of life, and school experiences	US	51 participants<br>8-17 y.o.<br>Cancer survivors	Divided into 2 groups (8-12 y.o. and 13-17 y.o.).<br>Mixed methods<br>Interviews<br>Open coding category-generating process.	Helpfulness and understanding of teachers<br>Academic performance<br>Peer interaction and acceptance<br>Homebound instruction<br>Importance of attending school
Cameron (2019)	The psychosocial interactions of Adolescent and Young Adult (AYA) cancer survivors and the possible relationship with their development	New Zealand	4 participants<br>16-19 y.o.<br>+ 6 participants<br>20-25 y.o.<br>Cancer survivors	Face-to-face interviews + one-year follow up interview.<br>Thematic analysis.	Personal privacy and sharing of information<br>Independence<br>Identify formation<br>Positivity<br>Acknowledgement v being treated normally<br>Support instead of supporting others
Choquette, Rennick & Lee (2015)	Back to School After Cancer Treatment: Making Sense of the Adolescent Experience	Canada	11 participants<br>13-17 y.o.<br>Cancer survivors	Face-to-face interviews.<br>Photo-elicitation.<br>Line-by-line, open and axial coding. Meaning-making theoretical model integrated as a guide.	Being on the right track to recovery<br>Bridging the two worlds<br>Establishing a new life at school
Christian, B. J.; D'Auria, J. P. (1997)	The child's eye: memories of growing up with cystic fibrosis	US	20 adolescents<br>12-18 y.o.<br>Cystic Fibrosis	Retrospective interviews<br>Life event line used.<br>Field notes also taken.<br>Open coding, axial coding.	Keeping secrets<br>Hiding visible differences<br>Discovering a new baseline
Cotter (2016)	The journey through school for children with cystic fibrosis : an interpretive phenomenological analysis	Ireland	4 participants<br>11-16 y.o.<br>Cystic Fibrosis	In-depth interview<br>IPA	Disruption to school life<br>Being in control<br>Reducing embarrassment<br>Friends supporting<br>Uncertainty<br>Goals and the future
D'Auria, Christian, Henderson & Haynes (2000)	The company they keep: the influence of peer relationships on adjustment to cystic fibrosis during adolescence	US	15 participants<br>17-22 y.o.<br>Cystic Fibrosis	Third study in a series of 3 qualitative studies.<br>Interviews.<br>Constant comparative method<br>Thematising and coding	Losing ground<br>Being out the loop<br>Finding a new company of friends<br>Fighting a never-ending battle

Dockett (2004)	"Everyone was really happy to see me!" The importance of friendships in the return to school of children with chronic illness	Australia	10 young people<br>5-16 y.o.<br>Unspecified chronic conditions	Face-to-face interviews.<br>Transcripts coded by 2 researchers.	Positive and negative aspects of friendships<br>Feeling excited<br>Feeling nervous<br>Disclosure<br>Difficulties in maintaining friendships
Ferguson & Walker (2014)	Getting on with life': resilience and normalcy in adolescents living with chronic illness	Australia	31 participants<br>10-18 y.o.<br>Unspecified chronic conditions	Longitudinal case studies<br>Informed by interviews of participants in 3 'waves' over 3 years (first one photo-elicitation)<br>Thematic coding<br>Both transcripts and materials (photos) analysed	Managing lives by focussing on opportunities<br>Influences, optimism and the future<br>Resistance to being seen as different<br>The role of school and peers<br>The interrelationship between normalcy and resilience
Fleischman K., Smothers, M.K, Christianson, H. F. & Carter, L. (2011)	Experiences of Adolescents with Type 1 Diabetes as They Transition from Middle School to High School	US	6 participants<br>14-15 y.o.<br>Type 1 Diabetes	Interviews, structured<br>Themes, then core ideas/ summaries, then cross-analysis.	Transition<br>Identity<br>Support<br>Friends and peers<br>Disclosure<br>Self-care
Forgeron, Evans, McGrath, Stevens & Finlay (2013)	Living with difference: Challenges to friendships for adolescents with pain	Canada	16 participants<br>14-18y.o.,<br>Chronic pain	Interviews<br>IPA	Rethinking the self with pain'<br>Integrating pain into the self<br>Understanding and sharing about chronic pain:<br><br>'Rethinking friendships'<br>Distancing the self<br>Needing a different quality in close friendships
Fottland (2000)	Childhood Cancer and the Interplay between Illness, Self-evaluation and Academic Experiences	Norway	8 school children 11-19 y.o.<br>Cancer survivors	Semi-structured interview<br>Young people interviewed individually and then parents introduced together and, lastly, hospital staff interviewed in groups.<br>Thematic	Attainment<br>Attendance<br>Lack of confidence<br>Peers<br>Catching up on social, physical and recreational activities a challenge
Gabe, Bury & Ramsay (2002)	Living with asthma: The experiences of young people at home and at school	UK	55 participants<br>11-16 y.o.<br>Asthma	Interviews<br>Thematic analysis	Experiencing asthma<br>Explaining asthma<br>Management of asthma
Gathercole (2017)	The educational experiences of children with cystic fibrosis	UK	5 participants<br>9-17 y.o.<br>Cystic Fibrosis	Online semi-structured interviews<br>Option for photovoice<br>Mixed analytical methods (thematic)	Being me first<br>Balancing treatments and school activities<br>Staying well at school<br>Knowing about CF<br>CF impacting learning<br>Educational support<br>Negotiating CF alongside adolescence

Glasson (1995)	A descriptive and exploratory pilot study into school re-entrance for adolescents who have received treatment for cancer	UK	5 adolescents<br>12-16 y.o.<br>Cancer	Semi-structured interviews.<br>Grounded theory, exploratory and descriptive.<br>Analysed emerging conceptual categories.	Disruption<br>Behind in school work<br>Normality
Holley, Walker, Knibb, Latter, Liossi, Mitchell, Radley, Roberts (2018)	Barriers and facilitators to self-management of asthma in adolescents: An interview study to inform development of a novel intervention	UK	28 participants<br>12-18 y.o.<br>Asthma	Focus groups and interviews<br>Inductive thematic analysis	Forgetting treatment<br>Managing medication<br>Knowledge<br>Difficulty concentrating<br>School staff, understanding and support at school
Holmstrom & Soderberg (2021)	The lived experiences of young people living with type 1 diabetes: A hermeneutic study	Sweden	10 participants<br>13-18 y.o.<br>Type 1 Diabetes	In-depth, open-ended, qualitative interviews.<br>Thematic analysis.	Living a transformed and re-organised everyday life<br>Feeling new emotions in the body<br>Living a governed everyday life<br>Being affected as a person<br>Being met with understanding and support<br>Informing about diabetes is important<br>School can be problematic
Kime (2014)	'Join us on our journey': exploring the experiences of children and young people with type 1 diabetes and their parents	England	116 participants<br>6-25 y.o.<br>Type 1 Diabetes	3 year multisite study. Nine acute trusts across Yorkshire and the Humber region.<br>Talking groups (term coined by the young people) separated by age (6-11, 12-14, 15-17, 18-25).<br>Thematic analysis.	Diabetes care<br>Education<br>Communication and support<br>School<br>Transition
Kuntz, Anazodo, Bowden, Sender & Morgan (2019)	Paediatric Cancer Patients' Treatment Journey: Child, Adolescent, and Young Adult Cancer Narratives	US	30 participants<br>10-25 y.o.<br>Leukaemia, Solid Tumours	Semi-structured interviews.<br>Transcripts analysed in detail by each member of team.<br>Thematic analysis. Peer evaluations.	Activity challenges<br>Disconnection from school:<br><br>Importance of storytelling<br>Communication challenges<br>The value of altruism towards patients in the future
Kyngas (2004)	Support network of adolescents with chronic disease: adolescents' perspective	Finland	40 participants<br>13-17 y.o.<br>Asthma, Epilepsy, Juvenile Rheumatoid Arthritis, Type 1 Diabetes	Interviews dealt with 2 topics: everyday life with a chronic disease and informant's support network.<br>Content analysis.	Peers:<br>Fellow sufferers<br>Friends without chronic condition<br><br>School:<br>Sometimes used condition as an excuse<br>Comfort in nurse knowing<br>Acceptance from peers v. teasing and causing trouble

Lakeman (2021)	The school experiences of young people with a chronic health condition : an interpretative phenomenological analysis	UK	5 participants<br>13-16 y.o.<br>Type 1 Diabetes, Epilepsy, Cerebral Palsy, Mitochondrial Genetic disease	Semi-structured interviews, creative task to prepare.<br>IPA.	Autonomy<br>Relationships and belonging<br>Navigating the School Day<br>Emotional Wellbeing at School<br>Planning for the Future
Li, Lopez, Chung, Ho & Chiu (2013)	The impact of cancer on the physical, psychological and social well-being of childhood cancer survivors	China	15 participants<br>8-16 y.o.<br>Leukaemia, Lymphoma, Brain Tumour, Osteosarcomas, Kidney Tumour, Germ-cell Tumour	Semi-structured interview<br>Content analysis<br>Peer debriefing	For most, some impact on academic performance and pay extra efforts to keep up
Lightfoot, Wright & Sloper (1999)	Supporting pupils in mainstream school with an illness or disability: young people's views	UK	33 participants<br>11-16 y.o.<br>Unspecified chronic condition, physical disability	Semi-structured interviews<br>Framework analysis	Absence<br>Exclusion<br>Relationships with teachers<br>Relationships with peers
MacMillan, Kirk, Mutrie, Moola & Robertson (2015)	Supporting Participation in Physical Education at School in Youth with Type 1 Diabetes: Perceptions of Teachers, Youth with Type 1 Diabetes, Parents and Diabetes Professionals	Scotland, UK	16 participants<br>7-14 y.o.<br>Type 1 Diabetes	Interviews for young people (also focus groups with teachers, FG and int with health care professionals).<br>Constructivist thematic analysis.	Differences between primary and secondary schools<br>Areas requiring address in all schools<br>What teachers can do to help accommodate youth with type 1 diabetes<br>What schools can do to help accommodate youth with type1 diabetes
Newbould, Francis & Smith (2007)	Young people's experiences of managing asthma and diabetes at school	UK	69 participants<br>8-15 y.o.<br>Asthma and Diabetes	Interviews with young people (and separate interviews with parents). .<br>Coding and thematising	Access to medication whilst at school<br>Use of medication at school<br>Impact on regimen<br>Role of teachers and 'informed friends'<br>Exercise<br>School trips<br>Being different
Pini, Gardner & Hugh-Jones (2019)	How and Why School Is Important to Teenagers with Cancer: Outcomes from a Photo-Elicitation Study	UK	12 participants<br>13-16 y.o.<br>Leukaemia, Lymphoma, Soft Tissue Sarcoma or Bone Tumor	Photo-elicitation interviews 2, 6, and 9 months post-diagnosis.<br>IPA.<br>9 participants completed 3 interviews, 3 completed 1.	Falling behind<br>Controlling the story<br>Conflicting priorities<br>Maintaining educational pace<br>A paused life?<br>Finding a way to be the same but changed<br>Legacy of missed schooling

Pini, Gardner, Hugh-Jones (2016)	How teenagers continue school after a diagnosis of cancer: experiences of young people and recommendations for practice	UK	12 participants<br>13-16 y.o.<br>Leukaemia, Lymphoma, Soft Tissue Sarcoma, Bone Tumour	Longitudinal, photo-elicitation study with interviews over three time points.<br>1 interview in first 2 months of diagnosis, one approx 6 months post diagnosis, one at approx 9 months post diagnosis.<br>IPA	Regulation and calibration<br>Regulatory importance of exams and grading<br>Belonging to the school community<br>Damaging effect of miscommunications<br>Changing peer dynamics and norms<br>Adapting to altered appearances<br>Special consideration v normality
Pini, Hugh-Jones, Shearsmith, Gardner (2019)	What are you crying for? I don't even know you' - The experiences of teenagers communicating with their peers when returning to school	UK	12 participants<br>13-16 y.o.<br>Lymphoma, Hodgkin’s Lymphoma, Osteosarcoma, A-plastic Anaemia or Acute Lymphoblastic Leukaemia	Photo-elicitation, interviews conducted at 3 time points during the year.<br>Following diagnosis of lymphoma, Hodgkin’s lymphoma, osteosarcoma, A-plastic anaemia, or acute lymphoblastic leukaemia.<br>IPA.	Approaches to telling<br>Lives becoming public property<br>Owning the story
Ragni Cappelletti, De Stasio, Tondo, Specchio, Vigevano & Gentile (2020)	The impact of epilepsy on adolescence: a quali-quantitative investigation using focus group	Italy	8 participants<br>15-20 y.o.<br>Epilepsy	Mixed methods:<br>qualitative, focus groups<br>quantitative, self-report questionnaire<br>Thematic coding	Peer relationships and acceptance<br>Autonomy<br>School as bad place to have a seizure<br>Lack of staff skills<br>Concern over academic achievement<br>Future
Secor-Turner, Scal, Garwick, Horvath & Kellerman Wells (2011)	Living With Juvenile Arthritis: Adolescents' Challenges and Experiences	US	7 participants<br>14-21 y.o.<br>Juvenile Arthritis	2 Focus groups, semi-structured interview protocol. 1 x 14-21 y.o. and 1x 22-29 y.o.<br>Descriptive content analysis	Challenges included playing sports/ participating, doing schoolwork and talking to teachers<br>Pain impeded ability to complete some work<br>Management of pain and exercising
Vera et al. (2015)	The lived experience of pain in adolescents diagnosed with cystic fibrosis	US	5 participants<br>13-19 y.o.<br>Cystic Fibrosis	Exploratory descriptive design.<br>Interviews exploring pain experiences within 5 domains: pain characteristics, activities, relationships, work/school life and healthcare team<br>Content analysis with team-based constant comparisons.	Negative impact of restrictions in daily life<br>Social life and emotional toll - missing school functions, social activities and sports<br>Disclosure to teachers
Wakefield, Puhl, Litt & Zempsky (2020)	"If it ever really hurts, I try not to let them know:” The use of concealment as a coping strategy among adolescents with Chronic Pain	Canada	18 participants<br>12-17 y.o.<br>Chronic Pain	5 focus groups of 3-5 adolescents.<br>Directed content analysis, inductive content analysis.	Avoidance of judgement<br>Avoidance of being a social burden<br>Desire to be treated normally<br>Social isolation<br>Cognitive burden

Wilkie (2012)	"Absence Makes the Heart Grow Fonder": Students with Chronic Illness Seeking Academic Continuity through Interaction with Their Teachers at School	Australia	11 participants<br>Year 10-12<br>Cancer,<br>Anorexia Nervosa,<br>Conversion Disorder, Renal Failure, Multiple Sclerosis and Cochlear implant complications	A qualitative collective case study of students and their teachers, involving observations, interview and questionnaire.	Teachers finding out<br>Teacher concern<br>Ambiguity of school and teacher responsibility
Winger et al (2013)	Sometimes it feels as if the world goes on without me': adolescents' experiences of living with chronic fatigue syndrome	Norway	18 participants<br>12-18 y.o.<br>Chronic Fatigue Syndrome	In-depth interviews	Being forgotten by friends, school teachers and family members<br>Teaching staff not understanding<br>Reduced school attendance<br>Feeling left behind by peers<br>Some positive aspects
Zhu & Van Winkel (2015)	Using an ICT tool as a solution for the educational and social needs of long-term sick adolescents	Belgium	8 participants<br>12-19 y.o.<br>Fibromyalgia, CVS, CRPS, Vascular<br>problems, Hodgkin's,<br>Operation leg<br>extension, Depressive symptoms, Auto-immune disease	Online survey (young people) and interviews (young people and parents separately)	maintaining connection with school<br>Peers and social contact<br>School community
