# Supplementary material for: School lives of adolescent school students living with chronic physical health conditions: a qualitative evidence synthesis
Source: Arch Dis Child. 2022 Dec 2;108(3):225–9. doi: 10.1136/archdischild-2022-324874 (PMC9985755; doi:10.1136/archdischild-2022-324874)
Supplement: Supplementary data [file archdischild-2022-324874supp003.pdf]

Author / Date	Title	Was there a clear statement of the aims of the research?	Is a qualitative methodology appropriate?	Was the research design appropriate to address the aims of the research?	Was the recruitment strategy appropriate to the aims of the research?	Was the data collected in a way that addressed the research issue?	Has the relationship between researcher and participants been adequately considered?	Have ethical issues been taken into consideration?	Was the data analysis sufficiently rigorous?	Is there a clear statement of findings?	How valuable is the research?
An 2019	Difficulty in returning to school among adolescent leukemia survivors: A qualitative descriptive study	Yes	Yes	Yes	Yes	Yes	No	No	Yes	Yes	Yes
Bessell 2001	Children surviving cancer: Psychosocial adjustment, quality of life, and school experiences	Yes	Yes	Yes	Yes	Yes	No	No	Yes	Yes	Yes
Cameron 2019	The psychosocial interactions of Adolescent and Young Adult (AYA) cancer survivors and the possible relationship with their development	Yes	Yes	Yes	Yes	Yes	Yes	Yes	Yes	Yes	Yes
Christian 1997	The child's eye: memories of growing up with cystic fibrosis	Yes	Yes	Yes	Yes	Yes	Yes	No	Yes	Yes	Yes
Cotter 2016	The journey through school for children with cystic fibrosis : an interpretive phenomenological analysis	Yes	Yes	Yes	Yes	Yes	Unsure	Yes	Yes	Unsure	Yes

D'Auria 2000	The company they keep: the influence of peer relationships on adjustment to cystic fibrosis during adolescence	Yes	Yes	Yes	Unsure	Yes	No	Yes	Yes	Yes	Yes
Dockett 2004	"Everyone was really happy to see me!" The importance of friendships in the return to school of children with chronic illness	Unsure	Yes	Yes	Yes	Yes	No	No	Yes	Yes	Yes
Ferguson 2014	'Getting on with life': resilience and normalcy in adolescents living with chronic illness	Yes	Yes	Yes	Unsure	Yes	No	No	Yes	Yes	Yes
Fleischman 2011	Experiences of Adolescents with Type 1 Diabetes as They Transition from Middle School to High School	Yes	Yes	Yes	Yes	Yes	Yes	No	Yes	Yes	Yes
Forgeron 2013	Living with difference: Challenges to friendships for adolescents with pain	Yes	Yes	Yes	Yes	Yes	Yes	Yes	Yes	Yes	Yes
Fottland 2000	Childhood Cancer and the Interplay between Illness, Self-evaluation and Academic Experiences	Yes	Yes	Yes	Unsure	Yes	No	No	Yes	Unsure	Yes

Gabe 2002	Living with asthma: The experiences of young people at home and at school	Yes	Yes	Yes	Yes	Yes	No	Yes	Yes	Unsure	Yes
Gathercole 2017	The educational experiences of children with cystic fibrosis	Yes	Yes	Yes	Yes	Yes	Yes	Yes	Yes	Yes	Yes
Glasson 1995	A descriptive and exploratory pilot study into school re-entrance for adolescents who have received treatment for cancer	Yes	Yes	Yes	Unsure	Yes	No	Yes	Yes	Unsure	Yes
Holley 2018	Barriers and facilitators to self-management of asthma in adolescents: An interview study to inform development of a novel intervention	Yes	Yes	Yes	Yes	Yes	Unsure	Yes	Yes	Yes	Yes
Holmstrom 2021	The lived experiences of young people living with type 1 diabetes: A hermeneutic study	Yes	Yes	Yes	Yes	Yes	Yes	Yes	Yes	Yes	Yes
Kime 2014	â€˜Join us on our journeyâ€™: exploring the experiences of children and young people with type 1 diabetes and their parents	Yes	Yes	Yes	Yes	Yes	No	Yes	Yes	Unsure	Yes

Kuntz 2019	Pediatric Cancer Patients' Treatment Journey: Child, Adolescent, and Young Adult Cancer Narratives	Yes	Yes	Yes	Yes	Yes	Yes	Yes	Yes	Yes	Yes
Kyngas 2004	Support network of adolescents with chronic disease: adolescents' perspective	Yes	Yes	Yes	Unsure	Yes	No	Yes	Yes	Yes	Yes
Lakeman 2021	The school experiences of young people with a chronic health condition : an interpretative phenomenological analysis	Yes	Yes	Yes	Yes	Yes	Yes	Yes	Yes	Yes	Yes
Li 2013	The impact of cancer on the physical, psychological and social well-being of childhood cancer survivors	Yes	Yes	Yes	Unsure	Yes	No	Yes	Yes	Yes	Yes
Lightfoot 1999	Supporting pupils in mainstream school with an illness or disability: young people's views	No	Yes	Yes	Yes	Yes	No	No	Yes	Yes	Yes
MacMillan 2015	Supporting Participation in Physical Education at School in Youth with Type 1 Diabetes: Perceptions of Teachers, Youth with Type 1 Diabetes, Parents	Yes	Yes	Yes	Yes	Yes	Yes	Yes	Yes	Yes	Yes

	and Diabetes Professionals										
Newbould 2007	Young people's experiences of managing asthma and diabetes at school	Yes	Yes	Yes	Yes	Yes	No	Yes	Yes	Yes	Yes
Pini 2016	How teenagers continue school after a diagnosis of cancer: experiences of young people and recommendations for practice	Yes	Yes	Yes	Yes	Yes	No	Yes	Yes	Yes	Yes
Pini 2019	'What are you crying for? I don't even know you' - The experiences of teenagers communicating with their peers when returning to school	Yes	Yes	Yes	Yes	Yes	Unsure	Yes	Yes	Yes	Yes
Pini 2019	How and Why School Is Important to Teenagers with Cancer: Outcomes from a Photo-Elicitation Study	Unsure	Yes	Yes	Yes	Yes	Yes	Yes	Yes	Yes	Yes
Ragni 2020	The impact of epilepsy on adolescence: a quali-quantitative	Yes	Yes	Yes	Yes	Yes	No	Yes	Yes	Yes	Yes

	investigation using focus group										
Rennick&Lee 2015	Back to School After Cancer Treatment: Making Sense of the Adolescent Experience	Yes	Yes	Yes	Yes	Yes	Unsure	Yes	Yes	Yes	Yes
Secor-Turner 2011	Living With Juvenile Arthritis: Adolescents' Challenges and Experiences	Yes	Yes	Yes	Yes	Yes	Yes	Yes	Yes	Yes	Yes
Vera 2015	The lived experience of pain in adolescents diagnosed with cystic fibrosis	Yes	Yes	Yes	Yes	Yes	Yes	Yes	Yes	Yes	Yes
Wakefield 2021	"If it ever really hurts, I try not to let them know:" The use of concealment as a coping strategy among adolescents with Chronic Pain	Yes	Yes	Yes	Yes	Yes	Yes	Yes	Yes	Yes	Yes
Wilkie 2012	"Absence Makes the Heart Grow Fonder": Students with Chronic Illness Seeking Academic Continuity through Interaction with Their Teachers at School	Yes	Yes	Yes	Yes	Yes	No	Unsure	Yes	Yes	Yes

Winger 2014	'Sometimes it feels as if the world goes on without me': adolescents' experiences of living with chronic fatigue syndrome	Yes	Yes	Yes	Yes	Yes	No	Yes	Yes	Yes	Yes
Zhu 2015	Using an ICT tool as a solution for the educational and social needs of long-term sick adolescents	Yes	Yes	Unsure	Yes	Yes	No	Unsure	Unsure	Yes	Yes
