# Supplementary material for: School lives of adolescent school students living with chronic physical health conditions: a qualitative evidence synthesis
Source: Arch Dis Child. 2022 Dec 2;108(3):225–9. doi: 10.1136/archdischild-2022-324874 (PMC9985755; doi:10.1136/archdischild-2022-324874)
Supplement: Supplementary data [file archdischild-2022-324874supp004.pdf]

	Keeping up / catching up / missing out / looking forward	Identity	Relationships with peers	Normality and difference	Autonomy	Relationships with staff
An & Lee 2019	✓	✓	✓	✓		
Bessel 2001	✓		✓	✓	✓	✓
Cameron 2019	✓	✓	✓	✓	✓	
Choquette, Rennick & Lee 2015	✓	✓	✓	✓	✓	✓
Christian & D'Auria 1997	✓	✓	✓	✓	✓	
Cotter 2016	✓	✓	✓	✓		✓
D'Auria, Christian, Henderson & Haynes 2000	✓	✓	✓		✓	
Dockett 2004	✓	✓	✓	✓		✓
Ferguson & Walker 2014	✓		✓	✓		
Fleischman et al. 2011	✓		✓	✓	✓	✓
Forgeron et al. 2013	✓	✓	✓	✓	✓	✓
Fottland 2000	✓	✓	✓	✓	✓	✓
Gabe, Bury & Ramsay 2002	✓		✓	✓	✓	✓
Gathercole 2017	✓				✓	✓
Glasson 1995	✓	✓	✓		✓	✓
Holley et al. 2018	✓		✓			✓
Holmstrom & Soderberg 2021	✓	✓	✓	✓	✓	✓
Kime 2014	✓		✓	✓	✓	✓
Kuntz et al. 2019	✓		✓			
Kyngas 2004	✓		✓		✓	✓
Lakeman 2021	✓		✓	✓	✓	✓
Li et al. 2013		✓	✓	✓	✓	
Lightfoot, Wright & Sloper 1999	✓	✓	✓	✓	✓	✓
MacMillan et al. 2015	✓	✓	✓	✓	✓	✓
Newbould, Francis & Smith 2007		✓	✓	✓	✓	
Pini, Gardner & Hugh-Jones 2016	✓	✓	✓	✓	✓	
Pini, Gardner & Hugh-Jones 2019		✓	✓	✓	✓	✓
Pini, Hugh-Jones, Shearsmith & Gardner 2019	✓		✓		✓	✓
Ragni et al. 2020	✓	✓	✓	✓	✓	
Secor-Turner et al. 2011	✓		✓			✓
Vera et al. 2015			✓	✓		✓
Wakefield, Zempsky, Puhl & Litt 2021	✓					✓
Wilkie 2012	✓	✓	✓			✓
Winger, Ekstedt, Wyller & Helseth 2014	✓		✓			✓
Zhu & Van Winkel 2014	✓		✓	✓	✓	
