# Supplementary material for: School lives of adolescent school students living with chronic physical health conditions: a qualitative evidence synthesis
Source: Arch Dis Child. 2022 Dec 2;108(3):225–9. doi: 10.1136/archdischild-2022-324874 (PMC9985755; doi:10.1136/archdischild-2022-324874)
Supplement: Supplementary data [file archdischild-2022-324874supp005.pdf]

	Keeping up / catching up / missing out / looking forward	Identity	Relationships with peers	Normality and difference	Autonomy	Relationships with staff
An & Lee 2019	“I want to be a nurse, doctor, or a teacher, but I cannot be them all, because my school grades need to be very good for it” p.74	“I was very conscious of people, I thought people were looking at me, even though they weren't...” p.73	“When I didn't go to school and would meet up with my friends, should I say that we didn't have much to discuss, when they talked about things that I didn't know about, I felt a little alienated…” p.72	"...when my class was being punished, I got many exceptions. I was worried that if teachers treat me that way even though I could do those things too and it was okay, my friends would not like it" (15 y.o. p.73)		
Bessel 2001	“I keep quiet and don’t ask a lot of questions.” “I keep a low profile so the teacher thinks I understand.” “I do just enough to get by so I can stay with my friends.” P.354		"the ones that stick around even when you're sick"<br>... "thought I had a disease they could catch or that I had AIDS" (p.354)			“especially sensitive,” “caring,” and “like a grandmother”<br>“She always found a way to make me feelgood and didn’t treat me like I would break” p.352
Christian & D'Auria 1997				“When I do sports and things, I can look normal, but I have to try twice as hard to do it” … “and so when I play baseball next time, I'm not going to tell my coach I have it. So he won't treat me any different than the other kids” p.7<br><br>“., they're normal, because you can't define		

				what normal is because they're an alternate different definition of normal, but they're normal ..” p.9		
Cotter 2016	"When I ... get sick ... I can miss a lot of time off school, then ... I’m trying to catch up ... in second year ... I missed so much school and in transition year ... I was in hospital as well so I missed so much school then too"<br><br>"‘I’ve no problems with getting to classes ... If I had a problem I’d ask a teacher and they’d help out ... if I miss school ... there’s teachers in the hospital that I talk to ... I ... try catch up on notes from ... the [local] teachers ... so it’s grand"	‘It’s just the way I am. Like I don’t act as if there’s something wrong ... as if I’ve a big problem. I just don’t see the point in that. I’m just acting myself and act normal as if I don’t have it. Like it doesn’t affect me that way that I have to act, I feel like I have to act a certain way or act different because I have CF and they don’t’	“my friends are so good like ... if there’s something wrong they’ll come down to me, they’ll stay with me ... after school ... they’ll make sure I’m okay” (P2, 9)	“See, I don’t want [the local] teachers to treat me differently because I’m sick” (P1, 22)<br><br>“I suppose ... they just think I’m normal really. Luckily ... I don’t want to be treated differently. I just want to be treated the same” (P4, 9)<br><br>“I don’t think they see me as anything different. I think they just see me as what I am.”		“If I had a problem I’d ask a teacher and they’d help out” (P1, 20)
D'Auria, Christian, Henderson & Haynes 2000	"I never got to know people as well as I would have if I hadn’t missed so much school."		“She [best friend] still asks questions. She still doesn’t understand it and I’ve known her 12years. You can never		“It’s something you’re going to have to try and take care of. You’re going to have medicines to take every day, every	

	"I'm not at school as much as some of the others, so you're kind of out of the loop when you come back after 3 weeks. Who's seeing who, you know. That can change radically in 3 weeks"		completely know somebody until you are someone with CF."		time you eat.... There will be times when you are sicker than others. There will be times when you don't feel good but you're not sick, and that's when you have to push yourself. That's when you got to go and make yourself do something!"p.180	
Ferguson & Walker 2014	"... I really started missing my life because you know being in hospital and sort of you have a boundary. Yeah I started missing my friends and my school and my life at that point." (p.236)<br><br>"Well it was hard, it was really hard at the start [of the year] because like I just didn't want to be there [at school] because it was too hard like to get up out of bed and go there"<br><br>""you get back to school and you're			"Yeah I guess the fact that I can still do, let's say most normal things that people my age do and yeah I guess that's sort of a milestone" (p.238)		"I had a teacher, our science teacher actually, she had a sort of like a class about what my illness was about so she told my class-mates with pictures and diagrams and that kind of thing, just a whole lesson about what Acute Myeloid Leukaemia was and what was happening to me and that kind of thing so that was, yeah I thought that was nice." P.237

	really behind and you have<br>to try and catch up and everything””					
Fleischman et al. 2011 (No quotes)						
Forgeron et al. 2013	“I studied like, every night. I study for my exams for a month in advance, I tried so hard. I passed all my courses, but not with the marks that I wanted. I think if I didn’t have the pain, I would have definitely had way higher marks than that.” E118	“It’s like an invisible enemy that no one really sees”	“they just don’t understand it or want to understand it, they don’t care or something, they’re just mean." (p.118)<br><br>“Well those kids, they let me die an emotionally social death, they just skewered me. None of them talk to me, and it’s still like, it irks me and it’s very painful…” “So we were very, very close, and then I was completely alienated." (p.120)<br><br>“you can tell that people are kind of like, uneasy around it or they don’t fully understand. They want to ask, but they don’t, and sometimes I’ll sort of bring it up to answer their	“I think I’d like them to react as normal as they could, even if I couldn’t do certain things or go certain places. I’d want them to act the same way.” E121		“This one teacher calls me the elevator mafia. And it doesn’t matter how many times I talk to him or that the guidance counselor talks to him, like he thinks he’s being funny and like that’s great and wonderful, but you have to be able to differentiate funny from mean. Like I don’t want to hear it.” E118

			questions. But it always kind of seems like a downer, and it's always about the pain." E117			
Fottland 2000	"I got a little spoiled, perhaps, I got to do whatever I wanted to, more or less, see, so I was dumb enough to use that quite a few times. Sure, I did some homework, like, but I got off easier than the others, y'know." P.263		"I'm always a bit afraid of how the others will react to what I say and do" p.263	"I didn't want to be different at school. That's why I didn't want any special aids, either, see… I didn't want any special assistance either, because I don't want to be worse than the others. When they don't need help, then I don't need help. I wanted to do everything the same as the others" p.267		
Gabe, Bury & Ramsay 2002	"You feel frustrated when you see this bunch of twenty people running off ahead and you think ''Why can't I do that?'' It would be nice to catch up with them once, but no." (p.1624)		"Yes, I am used to it now. I have been sent out of classes, I have been sent out of an exam because I have been annoying people." (p.1629)	"I just make myself do it, to show them just because I have got asthma I am no different"	"I have two or three of them. I keepone in my schoolbag and one at home. " (I.46)	
Gathercole 2017	"Erm, it doesn't really affect me that much...not really. I do everything I want to do"<br><br>"So, it never stopped me doing anything I		"sometimes I'm bothered(about doing treatment)because it means I don't get to see my friends. But then the other way, I know it's going to help	"I want to be seen as normal. I'm a normal person" p.106	"Sometimes if I need to go to the toilet and it's like...at my high school they don't let you go to the toilet during lessons" p.119	"she had to come in and like speak to a load of my teachers and staff that I had it. So that helped a lot."<br><br>"'School should probably know more

	really wanted to do" p.108<br><br>"They could like send work when you ask for it so you don't fall behind"p.135		me with my health" p.149			about it (CF) and what it does" p.127
Glasson 1995	"didn't know where to begin"<br>"didn't know how to catch-up" p.776	"lost my hair" "wore a wig"<br>"felt like a wally"<br>"just didn't feel right" p.776	"friends were brilliant" "helped me back into it" "there if I needed them". (p.756)<br><br>"learnt who my real friends were" p.776			
Holley et al. 2018						"It helps when the teachers know and they do trust you that you are late to class because I had to stop to take my inhaler"p.950
Kime 2014					"I was invited to a carb-counting class to help me understand how to read labels and be confident with carb-counting. This class was really helpful" (p.26)	"Teachers complain about me having to have snacks and have drinks and go to the toilet" (p.27)
Kuntz et al. 2019	"I write a journal on my computer and keep track of the daily 'every-thing' that happens. I have been doing this for the past month. I want to look at it in the end and see just					

	what I went through. In the future, I want to go to medical school, so I can look back on this experience to help me for in my essay" p.45					
Kyngas 2004			"some classmates always support me, even though some others tease me, for example, because I have to take my medication. The supporters remind me of how important it is to take my medication and also tell the teasers that they do not understand the importance of medication" (p.291)			"…it is safe to know that if I got such bad hypoglycemia that I cannot help myself, the school nurse will know what to do…"
Lakeman 2021	"I can't really concentrate" "I missed a lot of time" "Going to try to (--) not involve T1D in my future" "when I go to uni the big worry is around making sure that I've got the right support and the right accessibility"	(lots from author but not many direct quotes to support this)	"they push me towards to being friends more with disabled people" "I couldn't sit with my friends at lunch"	"…don't want to be treated differently to everyone else but sometimes I need to be treated differently"	"They feel that I can't manage it myself"<br><br>"My management was fine. There wasn't an issue. At all. But all of a sudden, she* made me miss a lesson which I liked. She* made me miss my break and this happens a lot." (Line 261) *she= school nurse	"None of the teachers are really helpful"<br><br>"I mean I've got a teacher at the moment who is also a diabetic too so if I am in her class and my pump stops or something, she'll know that I've got to do stuff and

	"it's quite hard to catch up with work as there is quite a lot, a lot to do at home"					she doesn't mind." (Line 259-261)
Lightfoot, Wright & Sloper 1999	"I miss enough of it when I'm really poorly and I just enjoy it when I'm there. So if I've got a bit of a headache I don't tell anybody...I have offdays but I don't really want to come home" p.272 Some of the teachers, if I asked them to explain it they would just say it's your own fault for not being here and I'd say `well it ain't my fault'...I would like more help with the work." P.273		"I get picked on, pick, pick, pick, pick, pick, and I get called the horriblest names and when I go off crying people go and tell (the deputy head), she doesn't do anything at all. I can't really do anything"p.277			"teachers couldn't understand that, they just thought I was not bothering or sometimes they didn't try and explain the work to me and they'd say `Oh, you know what you are doing'"p.273<br><br>was upset because I thought, well, all the teachers know, but they don't seem to care... I said `I can't do this and if you make me do it I'll be seriously poorly'. But they still made me do it." P.274<br><br>"The more people who know the less mistakes are made...ignorance is the root of all the problems I've had, it's people with a lack of knowledge that have made it difficult" p.275

Li et al. 2013	"I find difficulty in paying attention to what I am doing. In the past, I could study 6 h per day. After recovery, I am easily distracted by other things. I could hardly concentrate on my study."p.217 "I was a member of a football team at school. Now, I am no longer able to join the team because of the decrease in physical strength and endurance." "The impact cancer had on me was on the learning side. I had to make extra efforts in order to catch up with my studies after my remission." P.217		"After remission, I feel the age gap between my classmates. The main reason is that I haven't hung out with them for a long time. We cannot understand each other as we have different growing experience."p.217			
MacMillan et al. 2015 (very few YP quotes)						"I do tell them [teachers] but they don't actually realise, if I'm actually going to be low but I do tell them...Once eh, eh I

						had my lunch, I was low before it. Had my lunch, they test me after it, I was...well high, of course that was supposed to happen...They didn't know that. So then they had to make me run about and that...affect[ed] me to go low again...I don't think they realised how high it could go." (p.11)
Newbould, Francis & Smith 2007					“"We are meant to put our hand up and ask Miss for our blue(inhaler) but then I'd have to walk all the way to the office to get it. So my Mum she said to me just to keep it in my pocket or my pencil case and just use it when I need it.”p.1079	“... my friends said Mr Simms was just sort of staring and he didn't move" (p.1079) “but Mum went to the school and Mrs White also came to our house and she taught her all about what I need and what to do when I go high and low and stuff”p.1080
Cameron 2019		“the girl with cancer” “they might still think of me as the	“I lose quite a lot of friends because they'd say they were my friends and then	“…they still treated me like I was the same person and, just being treated like I was.. like		

		person with cancer but I'd like to think that I've kind of broken away from that now, and I'm now just me… Lisa 2.0.You know? Lisa post - cancer" p.62	they'd just talk about me behind my back, or be too scared to talk to me"p.65	I wasn't any different… was really helpful for me." P.65		
Pini, Gardner & Hugh-Jones 2016	"Because if they look back on that and then saw that [bad report] and not knowing what had gone on that year thinking in year 9 'oh she messed about, she got messy, she was predicted an F and now a U in history' it wouldn't have been very fair on me."	"...people obviously are going to say 'oh I wonder if she's got a wig on' because everybody would do that, I' d do that, just wonder." "sometimes I can just dump my crutches down and I'll walk, so then I think people are like, 'what an attention seeker!' kind of thing."	"I just needed her there! To be able to give me that confidence, because she's in a lot of my lessons as well so she's just. she's the strong one really!"	"I wanted them to not be exactly the same with me, I wanted them to sort of. sort of be responsible, normally they're quite crazy and weird. Like not feel sorry for me or anything but be more thoughtful but still be their normal selves."	"It was quite, not annoying but, I don't know, you just kind of wanted to be left alone sometimes and they were always asking, I don't know, like yeah, always like making sure you're okay, which is a good thing but it just, after a while it kind of gets a bit, I don't know, like the everyday thing."	"Well the sheet that I've been given has always got a contact list on. Which doesn't really mean that they're very helpful, it just means that they've got in contact!"

Pini, Gardner & Hugh-Jones 2019	"I think maybe like the concentration side of things. I'm a bit nervous about that actually, sort of sitting in a classroom listening for, working. I think it could be quite, not difficult, but a challenge" (p.13)	"'why are you crying? It's not you, it's my hair, I'm not bothered, it's just hair" (p.12) "That point was like, that was a good point for me because I had the confidence to actually go to school without my wig on." 12	"They [healthy peers] don't know what it's like and they don't know what pain you go through" (p.8)	"just want to been seen as me being normal" p.11	"get my body how I want it to be" "forget it [cancer]"	
Pini, Hugh-Jones, Shearsmith & Gardner 2019			"they all just asked, everybody asked me, what's that, what is it and I didn't know so I just said, I don't know but everybody asked me it, virtually everybody and it just got really irritating and annoying, you know" "So... yeah that first day back... people that I didn't even talk to normally they just started crying, and it was like 'what are you crying for? I		"So telling them I'd just say... I wouldn't tell them in front of everybody else, I'd tell them sort of like two or three people at a time and things and just say 'look I've got cancer and I will tell you everything that I could' and they were like 'oh right... are you going to beat it?', 'yeah, of course I am! What do you think?!"	

			don't even know you'"			
Choquette, Rennick & Lee 2015	"During my treatment, I didn't talk to anyone from school. No one. School was, like, gone. Like it was another world. Like the hospital was the new school, like my second world" (p.6)	''When I look at myself in the mirror, it's like I do not recognize myself.''	"I didn't know if [my friends] were gonna react like, 'oh, that guy had cancer, I don't want to get it.' They probably didn't know it was not contagious..." (p.4) "Just walking around the halls and everyone just staring at you, not talking, just feeling like [crap]. You know, everyday, I did not want to, like, go to school." (p.4) ''struggle to rebuild friendships'	'You feel like you're special, you're different. It's like you're someone else, not like everyone around you, 'cause you need more extra care or, like, attention so it didn't feel right.''	"I want to do this by myself, you know. It's my battle… I want to take this one for myself, but always she asked.''	
Holmstrom & Soderberg 2021		"It felt like I was regarded as a completely different person in school almost like as I was a disease..."		"It felt like I was regarded as a completely different person in school almost like as I was a disease." (p.6)	" do not feel safe at school.... I'm not sure people would notice if I got a hypo...." (p.6) – autonomy because reliant on self-management?	"It was strange that no one cared... it was like no one was responsible and no one cared and the headmaster thought it was so much fuss with me and told Mum not to enlarge problems." (p.7)

						“At first, Mum was in school with me and then she came to school for lunch and helped me with the insulin. Then Mum taught the teachers how to help me manage with blood sugar and all”
Secor-Turner et al. 2011	I play football there. And for me, I can’t play in all the games.I have to like go in a couple plays, sit out a couple plays, take my medicine. And they like, why you just not stayin’ in?.Because I have to do this so I could go in for the next round or do something else. Cause if I don’t do this, then my bones start achin’, swell up, and I’m in the hospital. So I’d rather take my medicine and sit		‘Having your friends understand what you’re going through.’ (= a challenge)			"like I did really bad in the sit-n-reach last year. And my teacher got really mad at me and then she told me that I needed an attitude adjustment, cause I was saying that I was sore whenever I was in gym or whatever. She was yelling at me and that was before I knew that I had arthritis. And then we told her about it, and she’s like, oh, whatever." (p.305)

	out a couple plays than go straight to the hospital" (p.305)					
Dockett 2004			They [peers at school] worry about these teeny little things ...I don't see them outside school...At school I just don't link up with any of the kids at school. I don't feel as if I'm a part ...I am just there." (p.31)<br><br>"It's personal. I only tell it to friends."p.30 (also autonomy?)		"It's personal. I only tell it to friends." (also peers?)<br><br>"I didn't really have the friends I have now back then ... I think they [previous friends] were too scared. They didn't know whether I would cry. They were scared to come near me because they didn't know whether I was sick." P.30	
Vera et al. 2015	"'I usually have tests like every other day... and I remember, 'Oh wait, I'm going to have to catch up on everything' and I'm like 'Blergh!'"		"'Most of my friends, like, they understand."			
Wakefield, Zempsky, Puhl & Litt 2020			"In my school I don't have like any friends and like if I did, they probably wouldn't	"when you have a 504 plan [school accommodations], sometimes you kind		"I wouldn't tell [my teachers] I'm having a whole bunch of pain... I would get on

			like if they probably want do a lot of stuff but like I can't like I can't go to the movies all the time because I can't walk around:::like then I would feel upset because like every day I come home and cry my eyes out because I have no friends."	off or get about what you have, or you try to ignore it, because you want to be as normal as possible and fit in with everybody, so you don't want to bring it up"<br><br>"you don't want to be treated like, kind of like, not like you're a patient, but you want to be treated as if you're like just regular."		the bad side of some teachers"
Wilkie 2012	I don't — I'm not good getting 'A<br>+<br>'s but I just want it to be a pass. That's all I want it to be.					"Keep in touch regularly"<br><br>"Just to try and stay in contact with them as much as you can, because they need to have their teacher there."<br><br>"I think just to, like don't pressure them but just stay in contact and, like I've found with all my

						teachers, they never, they just gave me the work and said if you're up to it, do it. There was never any pressure and that sort of helped."
Winger, Ekstedt, Wyller & Helseth 2014	"..actually I was only present at school, sitting on my chair in the classroom. Did nothing. Waiting to go home.." "No, then [in the future], I'm well, then I can do everything. Then I'm gonna catch up!"	"...I kind of separate between what I want and what my body wants, and so in a way I manage to distinguish between myself and my disease quite well"				
Zhu & Van Winkel 2014 (Most quotes about ICT tool)			"now my classmates saw me much more often, they trusted me more as they saw I was working hard" p.241			
Ragni et al. 2020	"I'm afraid not to find my place in the world, not to find a job"		"They distance themselves from you because you are different. Differences scare [people], it's a form	"They distance themselves from you because you are different. Differences scare [people], it's a	"Epilepsy is big trouble! Even at school, I can't go to the bathroom alone, somebody's got to come with me! I'm	

			of discrimination" (p.9)	form of discrimination" (p.9)<br><br>"They push you away because you are different."	forced to go to the toilet with the anxious thought that there is someone else there with me!"	
